# Breaking continuous potato cropping with legumes improves soil microbial communities, enzyme activities and tuber yield

**DOI:** 10.1371/journal.pone.0175934

**Published:** 2017-05-02

**Authors:** Shuhao Qin, Stephen Yeboah, Li Cao, Junlian Zhang, Shangli Shi, Yuhui Liu

**Affiliations:** 1 College of Horticulture, Gansu Agricultural University, Lanzhou, P.R. China; 2 CSIR–Crops Research Institute, Kumasi, Ghana; 3 Key Laboratory of Grassland Ecology System, Ministry of Education (Gansu Agricultural University), Lanzhou, P.R. China; Agriculture and Agri-Food Canada, CANADA

## Abstract

This study was conducted to explore the changes in soil microbial populations, enzyme activity, and tuber yield under the rotation sequences of Potato–Common vetch (P–C), Potato–Black medic (P–B) and Potato–Longdong alfalfa (P–L) in a semi–arid area of China. The study also determined the effects of continuous potato cropping (without legumes) on the above mentioned soil properties and yield. The number of bacteria increased significantly (p < 0.05) under P–B rotation by 78%, 85% and 83% in the 2, 4 and 7–year continuous cropping soils, respectively compared to P–C rotation. The highest fungi/bacteria ratio was found in P–C (0.218), followed by P–L (0.184) and then P–B (0.137) rotation over the different cropping years. In the continuous potato cropping soils, the greatest fungi/bacteria ratio was recorded in the 4–year (0.4067) and 7–year (0.4238) cropping soils and these were significantly higher than 1–year (0.3041), 2–year (0.2545) and 3–year (0.3030) cropping soils. Generally, actinomycetes numbers followed the trend P–L>P–C>P–B. The P–L rotation increased aerobic azotobacters in 2–year (by 26% and 18%) and 4–year (40% and 21%) continuous cropping soils compared to P–C and P–B rotation, respectively. Generally, the highest urease and alkaline phosphate activity, respectively, were observed in P–C (55.77 mg g^–1^) and (27.71 mg g^–1^), followed by P–B (50.72 mg mg^–1^) and (25.64 mg g^–1^) and then P–L (41.61 mg g^–1^) and (23.26 mg g^–1^) rotation. Soil urease, alkaline phosphatase and hydrogen peroxidase activities decreased with increasing years of continuous potato cropping. On average, the P–B rotation significantly increased (p <0.05) tuber yield by 19% and 18%, compared to P–C and P–L rotation respectively. P–L rotation also increased potato tuber yield compared to P–C, but the effect was lesser relative to P–B rotation. These results suggest that adopting potato–legume rotation system has the potential to improve soil biology environment, alleviate continuous cropping obstacle and increase potato tuber yield in semi–arid region.

## Introduction

Potato (*Solanum tuberosum* L.) is one of the staple food crops in the world. In northern China, potato has been the dominant crop, providing the main food source for the increasing human population [1]. In recent years, the tuber yield and water use efficiency (WUE) of potato in the semi–arid northwestern China has been greatly improved with the use of ridge–furrow and film mulching technology [2, 3]. The continuous planting with increased yield has resulted to important implications regarding tuber yield and quality [3–5]. Continuous potato cropping has been reported to cause a decline in soil fertility. For instance, a 3–year continuous potato cropping has been shown to decrease soil total nitrogen, phosphorus and potassium by 8%, 12%, and 9%, respectively compared to potato–maize rotation systems [5–8]. Also, continuous potato cropping increases the aggravation of toxic effect in root secretion such as palmitic acid and phthalic acid dibutyl, and also increases the pathogenic microbes (*fusaria* and *Mortierella*) which suppress plant growth and development.

Soil microbial diversity is important to sustainable agriculture because microbes can mediate many biochemical processes that support agricultural production. These processes include recycling of plant nutrients, maintenance of soil structure and degradation of agro–chemicals [9,10]. With increase in the years of continuous potato cropping, soil fungi harmful to root rhizosphere can increase, while bacteria and actinomycetes beneficial to plant roots can decrease significantly [11,12]. Consequently, the microbial population structure in soil becomes unbalanced with harmful microorganisms such as *Fusaria* and *Mortierella*, providing negative effect on plant root growth [13]. Furthermore, soil enzymes play a very important role in material cycle and energy conversion in the soil ecosystem [14]. Soil enzymes are important materials for catalyzing biochemical reactions, and provide an important indicator for measuring movement and strength of various soil biochemical processes [15]. However, enzyme activities can be restricted with continuous cropping. For instance, the activities of alkaline phosphatase, sucrase and urease were reduced with continuous potato cropping, which decreased potato yield and quality [5, 8, 13]. Therefore, it is imperative to explore possible approaches with which the obstacles associated with continuous potato cropping can be minimize and tuber yield and quality of potato improved.

Many studies have shown that crop rotation can help reduce or overcome the above–mentioned problems [16, 17]. Scientific and reasonable rotation systems can minimize continuous cropping obstacles, and the rotation of forage–crop has been shown to be an effective approach to achieve this goal [18, 19]. In recent years, due to feeding need for off–season forages, forage–crop rotation systems have developed rapidly in the northwest region of China [19]. Potato–leguminous forage rotation system has been shown to be of great significance for the improvement of soil fertility and enhancement of crop production in the Loess Plateau where soil and water erosion is severe [20]. Crop rotations with legumes can significantly increase the productivity of farmland system [21], improve economic benefits [22], and lower environmental footprints [23].

Besides crop rotation, soil tillage and fertility can affect the composition, diversity and functionality of soil microbial community, leading to changes of soil biochemical processes [24]. Furthermore, soil microorganisms play an essential role in the processes of organic matter decomposition and nutrient cycling, and thus affect soil fertility [24, 25]. Long term crop rotations with pastures can improve soil quality and contribute to a more sustainable use of soil resources compared to monoculture [26–30]. Crop and pasture rotations would maintain soil properties within acceptable limits and meet the goals of sustainable agriculture [29]. In the present study, we hypothesize that the use of potato–legumes rotation can improve soil biological properties and thus sustainably increase potato tuber yield to meet the increasing demand. However, little is known about potato–legumes rotation on soil microbial communities and enzyme activities. The objective of this study was to determine the effect of potato–legume rotation on soil microbial community characteristics, soil enzyme activities and tuber yield in semi–arid environment.

## Material and methods

### Site description

The field experiment was conducted in 2012 and 2013, respectively at the Dingxi Experimental Station (35°33′N, 104°35′E, elevation 1874 m a.s.l.) of Gansu Agricultural University. in Northwestern China. The experimental site had adeep soil layer, high water–storage capacity, wilting percentage at 7.3% and mid–level soil fertility. The aeolian soil is locally known as Huangmian soil [31]. The soil has total organic C value in 0–30 cm soil layer of 8.19 g kg^−1^ and readily oxidizable organic carbon content of 4.84 g kg^–1^. The average annual precipitation between 1970–2013 was 391 mm (Weather Bureau of Dingxi city, China). On average, about 54% of the annual rainfall occurs between July and September. Maximum daily temperature can reach 38°C in July, while minimum temperatures usually drop to –22°C in January. The annual average radiation is 5929 MJ m^–2^, and annual sunshine duration is 2477 hours.

### Experimental design

The study consisted of potato–legume rotation and continuous potato cropping administered in a randomized complete block design with three replicates. Experimental work of the potato–legume rotation included the following treatments: Potato–Common vetch (P–C), Potato–Black medic (P–B) and Potato–Longdong alfalfa (P–L). The continuous potato cropping is made of five time scales of potato cropping: i.e., continuous potato cropping for 1–year (CK1), 2–year (CK2), 3–year (CK3), 4–year (CK4) and 7–year (CK7). The three legumes used in the potato–legume rotations [Common vetch (*vicia sativa*), Black medic (*Medicago lupulina*) and Longdong alfalfa (*Medicago sativa*) are popular in the study area. Planting was done in April each year and plot size was 3 m × 2 m. The continuous potato cropping phase of this study ended in 2011 and the potato–legume rotation was introduced in 2012. Therefore the three legumes were planted in 2012 with seeding rates of 120 kg ha^–1^ for *Vicia sativa*, 45 kg ha^–1^ for *Medicago lupulina*, and 30 kg ha^–1^ for *Medicago sativa* and harvested in April 2013. After harvesting the legumes, all plots were planted with potato tubers at the density of 45400 plants ha^–1^ and harvested in October 2013.The seeds of legumes were provided by the Key Laboratory of Grassland Ecological System of Ministry of Education (Gansu Agricultural University).

### Measurements and methods

#### Soil samples collection

Soil microbial communities and enzyme activities were determined on two occasions, namely prior to planting the legumes in 2012 (continuous potato cropping, (CK)) and at harvest of legumes. As such the soil samples in the continuous potato cropping field were also collected prior to planting the legumes in April 2012. In the potato–legume rotation, soil samples were collected at harvest of the legumes in April 2013. Soil augers (6 cm diameter and 20 cm long) were used for soil sampling in the experiment. In each plot, eight soil cores (“W” sampling method) were collected to a depth of 20 cm. The sampled soil was divided into two parts; one part (fresh samples) was put into sterile ziplock bags after removal of large plant material. The samples were then transferred to the laboratory for measurement of soil microbial communities immediately. The other part of the sample was air–dried, ground, sieved (mesh size 1×1 mm^2^) to remove root fragments and stored at room temperature for determination of enzyme activities.

#### Microbial community

Quantity of bacteria, actinomycetes, fungi, aerobic azotobacters and anaerobic azotobacters were measured using the method described by [32] and [33]. Numbers of colony–forming units (CFU) (g^−1^) of fresh soil were measured in duplicate using serial dilutions of 10 g dry soil with distilled water. At each dilution, some samples were applied to Stephenson culture medium incubated at 26°C for 14 days to assess nitrifying bacteria. For denitrifying bacteria, the culture medium was peptone with agar in test tubes that were incubated at 25°C for 14 days [34–36].

#### Soil enzyme activity

Hydrogen peroxidase activity was determined using KMnO_4_ titration method; urease activity was measured using phenol sodium hypochlorite colorimetric method; and phosphatase activity was measured using disodium phenyl phosphate colorimetric method [37].

#### Tuber yield

The tuber yield per hectare (kg ha^–1^) was obtained by determining the tuber yield of each plot in each year at harvest. The tuber yield in the continuous potato cropping fields (CK) was determined in October 2011 and that of potato–legume rotation was determined in October, 2013.

### Data analyses

Data were analyzed using the mixed effect of the SPSS statistical analysis software (SPSS software, 17.0, SPSS Inst. Ltd., USA) with the rotation treatment as the fixed effect and replicate as random effect. Means comparison based on Duncan’s multiple range test (p<0.05). Due to significant year by treatment interactions for most of the variables evaluated in the study, the treatment effect was assessed separately for each year.

## Results

### Soil microbial communities

#### Continuous cropping effects on bacteria, fungi and actinomycetes

The effect of continuous potato cropping on bacteria, fungi and actinomycetes are presented in Table 1. Bacterial numbers (BNs) were significantly increased (p <0.05) by 124% and 86% in the 2 and 3–year continuous potato cropping fields respectively compared to the 1–year continuous potato cropping field. The maximum fungi numbers (FNs) were observed in the 4 and 7–year continuous cropping fields; it was, respectively, 87% and 48% more compared to the 1–year continuous cropping soils. Actinomycetes numbers (ANs) were significantly higher in the 3 and 4–year continuous cropping soils compared to the 1 and 7–year continuous cropping soils. Fungi numbers showed an increasing trend with increased in continuous cropping years, whiles bacteria numbers decreased in the 4 and 7–year continuous cropping fields. Actinomycetes numbers presented an increasing trend with an increase in continuous cropping years, except in the 7– year continuous cropping soils (Table 1).

**Table 1:** Soil microorganisms under continuous potato cropping.

Years of continuous cropping	Bacteria (×10^6^ CFU/g)	Fungi (×10^3^ CFU /g)	Actinomycetes (10^5^ CFU /g)
CK1	31.9 c	9.7 b	1.1 c
CK2	71.5 a	10.2 b	8.2 b
CK3	59.4 b	13.0 ab	14.9 a
CK4	35.4 c	14.4 ab	12.5 ab
CK7	42.7 c	18.1 a	5.9 c

Note: Different letters in the same column meant significant difference among treatments at p <0.05. CK1, CK2, CK3, CK4 and CK7 represent the values of 1–4 and 7 year continuous potato cropping soil.

#### Crop rotation effects on bacteria, fungi and actinomycetes

Potato–legumes rotation had significant effect (p <0.05) on bacteria, fungi and actinomycetes numbers (ANs) depending on treatment (Table 2). The number of bacteria had a consistent increase under P–B and to a lesser extent under P–L rotation. Bacteria numbers (BNs) were increased under P–B rotation by 78%, 85% and 83% in the 2, 4 and 7–year continuous cropping plots, respectively compared to P–C rotation. The P–L rotation had the highest fungi numbers (FNs) in the 1 and 2–year continuous cropping plots, whiles P–C rotation had the highest BNs in the 3 and 7–year continuous cropping plots. The fungi numbers recorded an increasing trend under P–B relative to the 1–year continuous cropping plots. On the contrary, the trend of FNs under P–C and P–L were not consistent. With exception of the 1–year continuous cropping plots, P–C rotation produced significantly lower actinomycetes numbers (ANs) compared to P–L rotation and to a lesser extent relative to P–B rotation (Table 2).

**Table 2:** Soil microorganisms under potato–legume rotation systems.

Years of continuous cropping	Crop rotation	Bacteria (×10^6^ CFU /g)	Fungi (×10^3^ CFU /g)	Actinomycetes (10^5^ CFU /g)
One				
	P–C	144.4 a	37.5 a	49.7 a
	P–B	128.1 ab	12.7 b	15.7 b
	P–L	124.4 b	38.7 a	27.5 b
Two				
	P–C	115.5 b	8.1 b	26.3 b
	P–B	205.7 a	17.0 a	48.2 a
	P–L	120.3 b	24.5 a	48.1 a
Three				
	P–C	107.1 a	45.3 a	31.7 b
	P–B	139.8 a	22.5 b	15.0 c
	P–L	130.1 a	28.3 b	51.9 a
Four				
	P–C	119.3 b	26.0 b	14.6 b
	P–B	221.2 a	43.0 a	17.6 ab
	P–L	180.2 ab	35.1 a	20.4 a
Seven				
	P–C	122.0 b	64.4 a	4.2 b
	P–B	223.1 a	33.2 b	5.7 b
	P–L	221.0a	4.4 c	20.1 a

Note: Different letters in the column meant significant difference among treatments at p <0.05. P–C, P–B and P–L refer to Potato–Common vetch, Potato–Black medic and Potato–Longdong alfalfa, respectively.

#### Continuous cropping effects on fungi/ bacteria ratio

**Fig 1:**
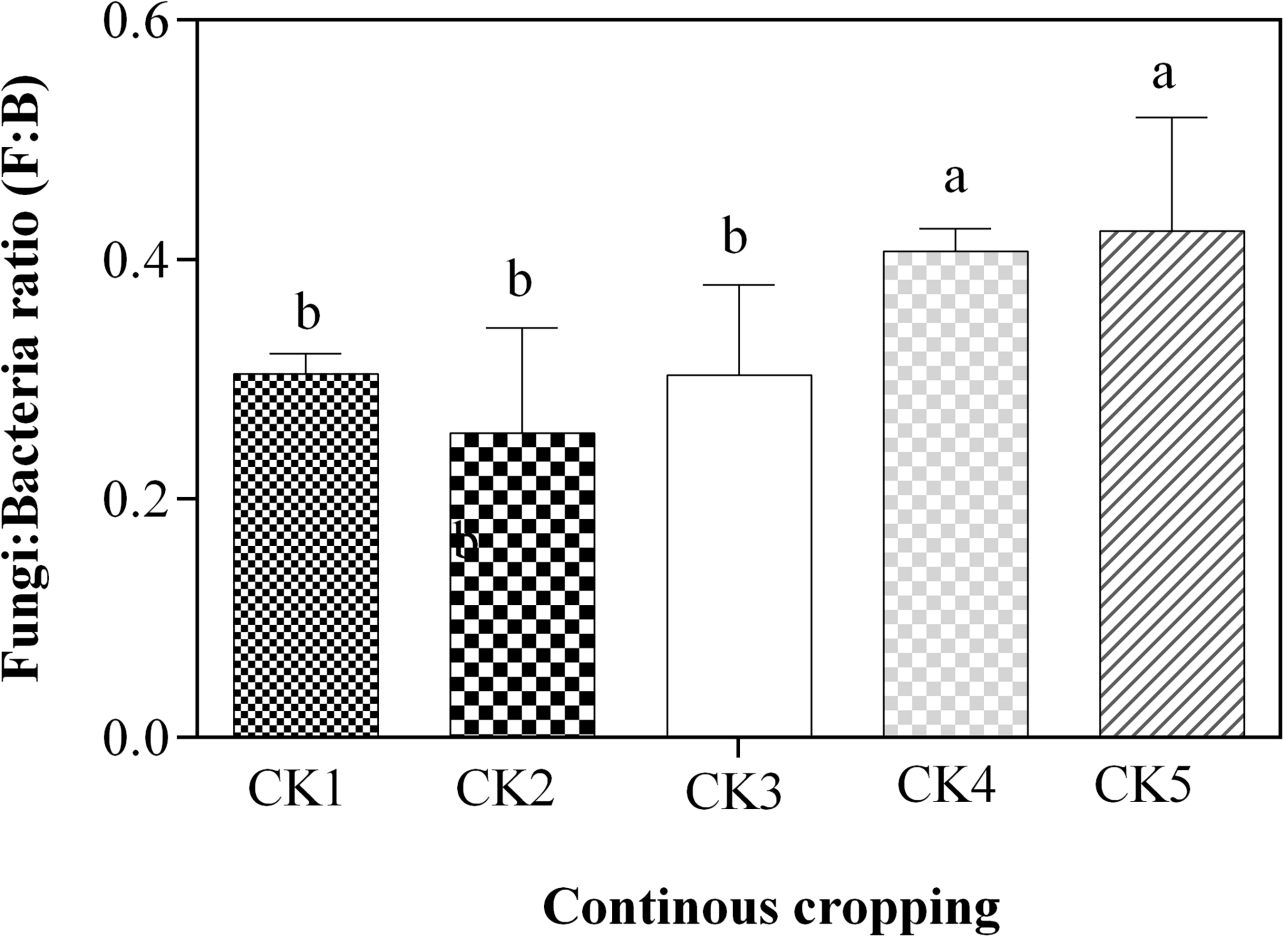
Effect of continous potato cropping on soil Fungi/bacteria ratio. Different letters denote statistically different values at p<0.05. Error bars represent the SE. Mean values ± SE (n = 3), and means comparison based on Duncan’s multiple range test (p<0.05).

Continuous cropping had significant effect (p <0.05) on fungi/bacteria ratio (Fig 1). The results showed an initial decrease in F: B ratio in the 2 and 3–year continuous cropping soils compared to the 1–year continuous cropping soils. However, the F: B ratio increased in subsequent years (4 and 7–year continuous cropping soils). The greatest F: B ratio was recorded in the 4 and 7–year continuous cropping soils and was significantly higher than that of 1–3 years continuous cropping soils.

#### Crop rotation effects on fungi/bacteria ratio

**Fig 2:**
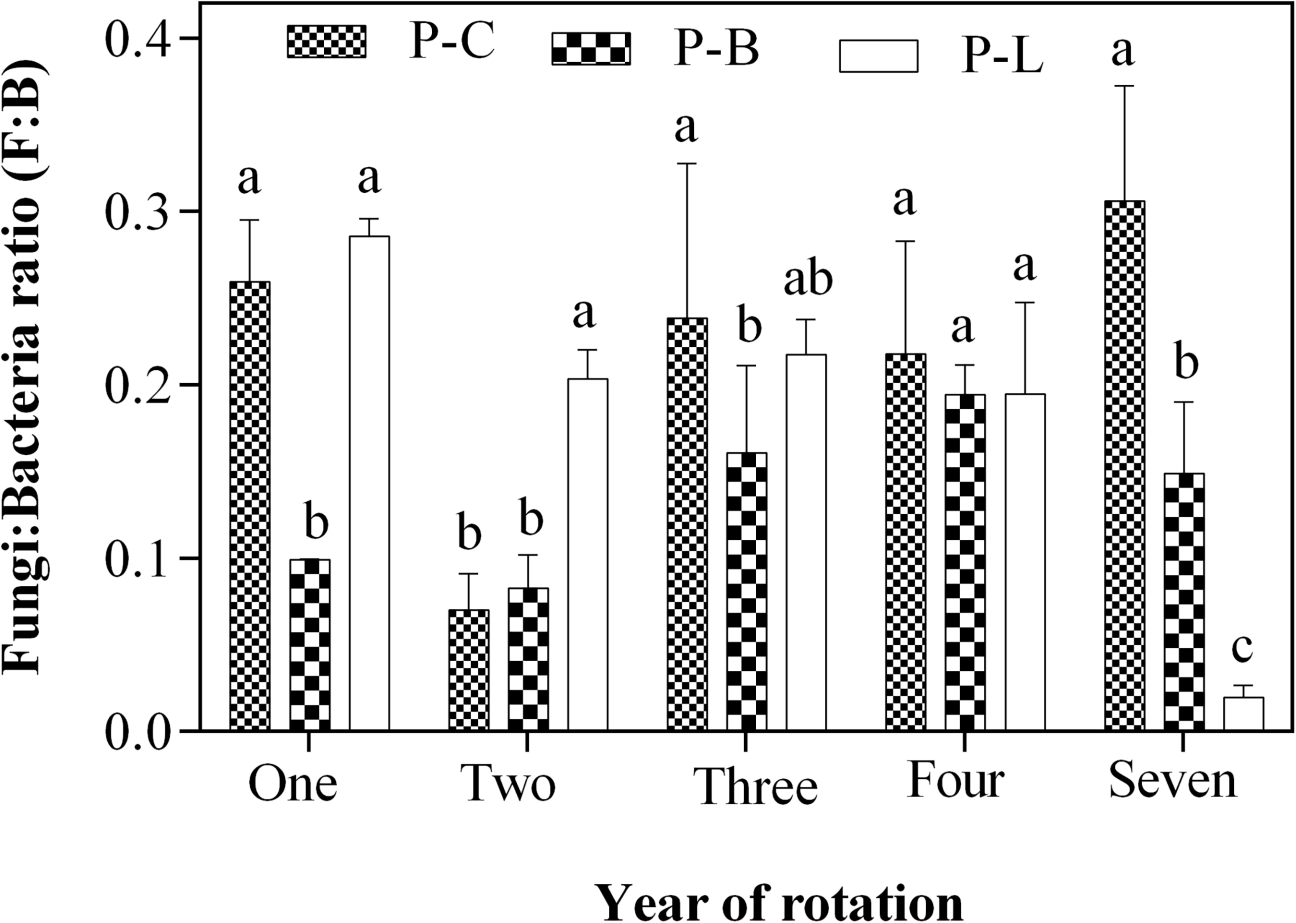
Effect of potato–legume rotation on soil Fungi/bacteria ratio. Different letters denote statistically different values at p<0.05. Error bars represent the SE. Mean values ± SE (n = 3), and means comparison based on Duncan’s multiple range test (p<0.05).

The trend of fungi/ bacteria ratio under various crop rotation systems was not consistent in all the years of this study (Fig 2). The P–B rotation had least F: B ratio in 1–year (0.099) and 3–year (0.161) continuous cropping soils, which were significantly lower than P–C (0.261 and 0.238) and P–L (0.286 and 0.218), respectively. The P–L rotation had the greatest F: B ratio of 0.204 and this was higher than P–C (0.0701) and P–B (0.0826) in the 2–year continuous cropping soils. Overall, the highest F: B ratio was found in P–C (0.218), followed by P–L (0.184) and then P–B (0.137).

#### Continuous cropping effects on azotobacters and nitrifying and denitrifying bacteria’s

Aerobic azotobacter increased with extension in potato continuous cropping years, except in the 2– year continuous plots (Table 3). In contrast, the quantity of anaerobic azotobacter reduced with the extension of potato continuous cropping years. The highest aerobic azotobacter was observed in the 3–year (87.23 10^4^ CFU /g), which was significantly higher than the other years of continuous cropping by 505% on average. Response of both nitrifying and denitrifying bacteria to continuous potato cropping was not consistent (Table 3). In comparison to the 1–year continuous cropping soils, nitrifying bacteria (NB) declined in the 2 and 7–year continuous cropping soils, and denitrifying bacteria (DB) decreased in the 4 and 7–year continuous cropping soils (Table 3). The highest nitrifying bacteria was found in the 3–year fields; it significantly increased by 63%, 124% and 152% compared to 1, 2 and 7–year cropping soils, respectively. Denitrifying bacteria was significantly higher (p <0.05) in the 2–year continuous cropping soils compared to 4 and 7–year continuous cropping soils.

**Table 3:** Azotobacter and nitrifying and denitrifying bacteria’s under continuous potato cropping.

Years of continuous cropping	Aerobic azotobacter (AA) (10^4^ CFU /g)	Anaerobic azotobacter (ANA) (10^4^ CFU /g)	Nitrifying bacteria (10^3^ CFU /g)	Denitrifying bacteria(10^3^ CFU /g)
CK1	25.48 c	11.53 a	0.51 b	1.09 ab
CK2	5.27 d	2.93 b	0.37 bc	1.76 a
CK3	87.23 a	3.43 b	0.83 a	1.61 a
CK4	62.23 b	3.50 b	0.82 a	0.82 b
CK7	30.54 c	2.78 b	0.33 c	0.44 c

Note: Different letters in the same column meant significant difference among treatments at p <0.05. CK1, CK2, CK3, CK4 and CK7 represent the values of 1–4 and 7 year continuous potato cropping soil.

#### Crop rotation effects on azotobacters and nitrifying and denitrifying bacteria’s

The effect of potato–legume rotation on aerobic and anaerobic azotobacter, nitrifying and denitrifying bacteria was significant (p <0.05) depending on treatment and cropping year (Table 4). The treatment effect on azotobacter, bacteria and the trend of change in response to cropping years were not consistent. The P–L rotation increased aerobic azotobacter by 26% and 18% in the 2–year and 40% and 21% in the 4–year continuous cropping soils compared to P–C and P–B rotations, respectively. The P–B had the highest aerobic azotobacter in the 2–year continuous cropping soils; an increase of 17% was observed compared to P–L rotation. Under P–B rotation, the anaerobic azotobacter had a significant increase of 66%, 92% and 217% on average in the 1, 2 and 4–year continuous cropping soils, respectively compared to P–C and P–L rotations. The nitrifying bacteria under P–B and to a lesser extent under P–C were significantly lower than that of P–L in the 1–4 year continuous cropping soils compared to P–L. But the P–B rotation had the highest anaerobic bacteria in 1, 2, 4 and 7– year continuous cropping soils compared to the other crop rotation systems (Table 4).

**Table 4:** Azotobacter and nitrifying and denitrifying bacteria’s under potato–legume rotation systems.

Years of continuous cropping	Crop rotation	Aerobic azotobacter (AA) (10^4^ CFU /g)	Anaerobic azotobacter (ANA) (10^4^ CFU /g)	Nitrifying bacteria (10^3^ CFU /g)	Denitrifying bacteria(10^3^ CFU /g)
One					
	P–C	77.46 b	11.99 b	0.85 a	18.95 b
	P–B	83.11 b	25.44 a	0.51 b	57.24 a
	P–L	97.75 a	18.72 b	0.87 a	56.15 a
Two					
	P–C	106.59 a	18.24 b	3.04 b	17.02 b
	P–B	106.79 a	31.17 a	2.93 b	37.40 a
	P–L	91.76 b	14.28 b	4.28 a	4.89 c
Three					
	P–C	69.52 a	24.07 a	1.71 b	35.36 b
	P–B	53.36 b	14.74 b	1.30 b	13.64 c
	P–L	63.66 ab	23.58 a	2.41 a	53.33 a
Four					
	P–C	91.48 b	23.63 b	1.65 b	23.63 a
	P–B	106.07 b	55.29 a	1.35 b	24.57 a
	P–L	128.01 a	11.24 c	2.37 a	18.93 b
Seven					
	P–C	83.15 a	18.80 a	3.76 a	19.22 b
	P–B	54.25 b	11.58 b	1.34 b	30.36 a
	P–L	60.78 b	15.43 b	1.81 b	11.55 c

Note: Different letters in the column meant significant difference among treatments at p <0.05. P–C, P–B and P–L refer to Potato–Common vetch, Potato–Black medic and Potato–Longdong alfalfa, respectively.

### Soil enzyme activity

#### Continuous cropping effects on soil enzyme activity

Soil urease, alkaline phosphatase and hydrogen peroxidase activities decreased gradually with increased years under continuous potato cropping (Table 5). The lowest Urease, alkaline phosphate and hydrogen peroxidase activities occurred in 2 and 7–year continuous potato cropping soils.

**Table 5:** Soil enzyme activity under continuous potato cropping.

Years of continuous cropping	Urease (mg g^–1^)	Alkaline phosphatase (mg g^–1^)	Hydrogen peroxidase (ml g^–1^)
CK1	18.12 a	18.99 a	17.95 a
CK2	15.05 a	8.19 b	14.13 ab
CK3	5.87 c	3.45 c	12.20 b
CK4	9.06 b	5.09 b	7.06 c
CK7	8.31 b	3.23 c	5.66 c

Note: Different letters in the same column meant significant difference among treatments at p <0.05. CK1, CK2, CK3, CK4 and CK7 represent the values of 1–4 and 7–year continuous potato cropping soil

#### Crop rotation effects on soil enzyme activity

Soil urease, hydrogen peroxidase and alkaline phosphatase activities were significantly increased in the 2–year cropping soils under the potato-legume rotation (Table 6). The rotation systems had significant effect (p <0.05) on soil urease, alkaline phosphate and hydrogen peroxidase activities depending on rotation and cropping year. The P–C rotation system had a better effect increasing soil urease activities, whiles P–B rotation systems had significant increase on alkaline phosphatase and hydrogen peroxidase activities.

**Table 6:** Soil enzyme activity under different potato–legume rotation systems.

Years of continuous cropping	Crop rotation	Urease (mg g^–1^)	Alkaline phosphatase (mg g^–1^)	Hydrogen peroxidase (ml g^–1^)
one				
	P–C	73.33 a	37.78 a	19.57 b
	P–B	71.61 a	21.79 b	25.19 a
	P–L	44.30 b	33.90 a	13.78 b
Two				
	P–C	77.56 a	21.30 a	22.88 a
	P–B	73.27 a	25.33 a	19.19 b
	P–L	42.07 c	18.62 b	17.82 b
Three				
	P–C	39.48 a	18.80 a	14.76 a
	P–B	20.58 c	20.17 a	15.03 a
	P–L	43.41 a	17.17 b	14.52 a
Four				
	P–C	51.60 a	32.70 a	12.00 b
	P–B	34.32 b	23.67 b	19.53 a
	P–L	38.87 b	21.67 b	14.55 b
Seven				
	P–C	36.88 b	27.98 b	11.87 a
	P–B	53.81 a	37.23 a	9.22 b
	P–L	39.41 b	24.96 b	10.39 b

Note: Different letters in the column meant significant difference among treatments at p <0.05. P–C, P–B and P–L refer to Potato–Common vetch, Potato–Black medic and Potato–Longdong alfalfa, respectively.

### Tuber yield of potato

#### Continuous cropping effects on tuber yield

**Fig 3:**
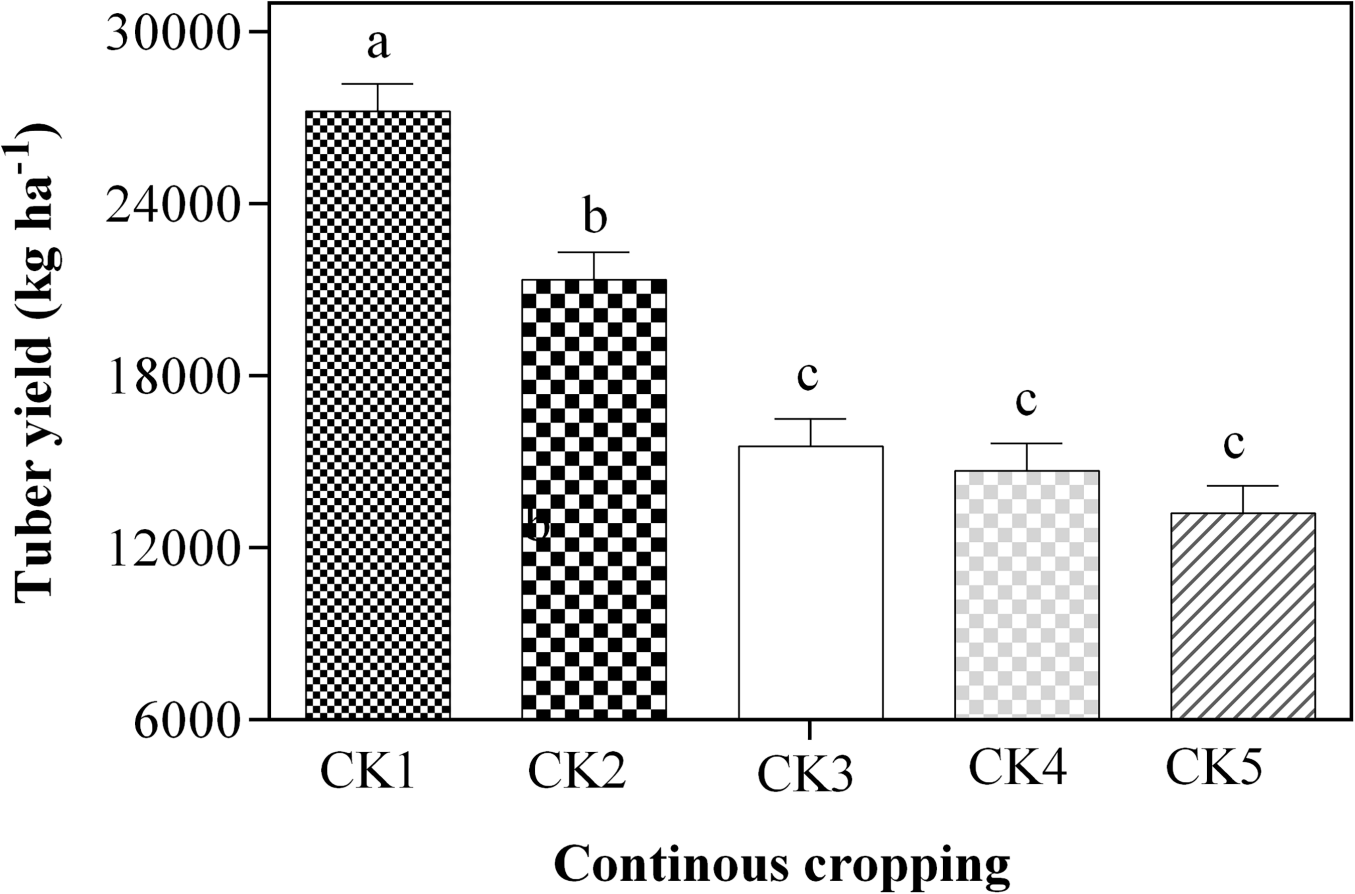
Effect of continous potato cropping on potato tuber yield (kg ha^–1^). Different letters denote statistically different values at p <0.05. Error bars represent the SE. Mean values ± SE (n = 3), and means comparison based on Duncan’s multiple range test (p <0.05).

The result on effect of continuous cropping on potato tuber yield is presented in Fig 3. The result was clear that, the tuber yield reduced with increased cropping years. The potato tuber yield had a significant decrease of 27%, 75% and 85% in the 2, 3 and 4–year continuous cropping soils, respectively compared to the 1–year cropping soils. Moreover, the tuber yield in the 2–year cropping soils was 21373 kg ha^–1^, which was significantly greater by 62% (13220 kg ha^–1^), 45% (14706 kg ha^–1^) and 37% (15553 kg ha^–1^) compared to 4, 4 nd 7 years, respectively. However, no significant differences (p <0.05) were observed in tuber yield between 3–7 years continuous cropping plots (Fig 3).

#### Crop rotation effects on tuber yield

**Fig 4:**
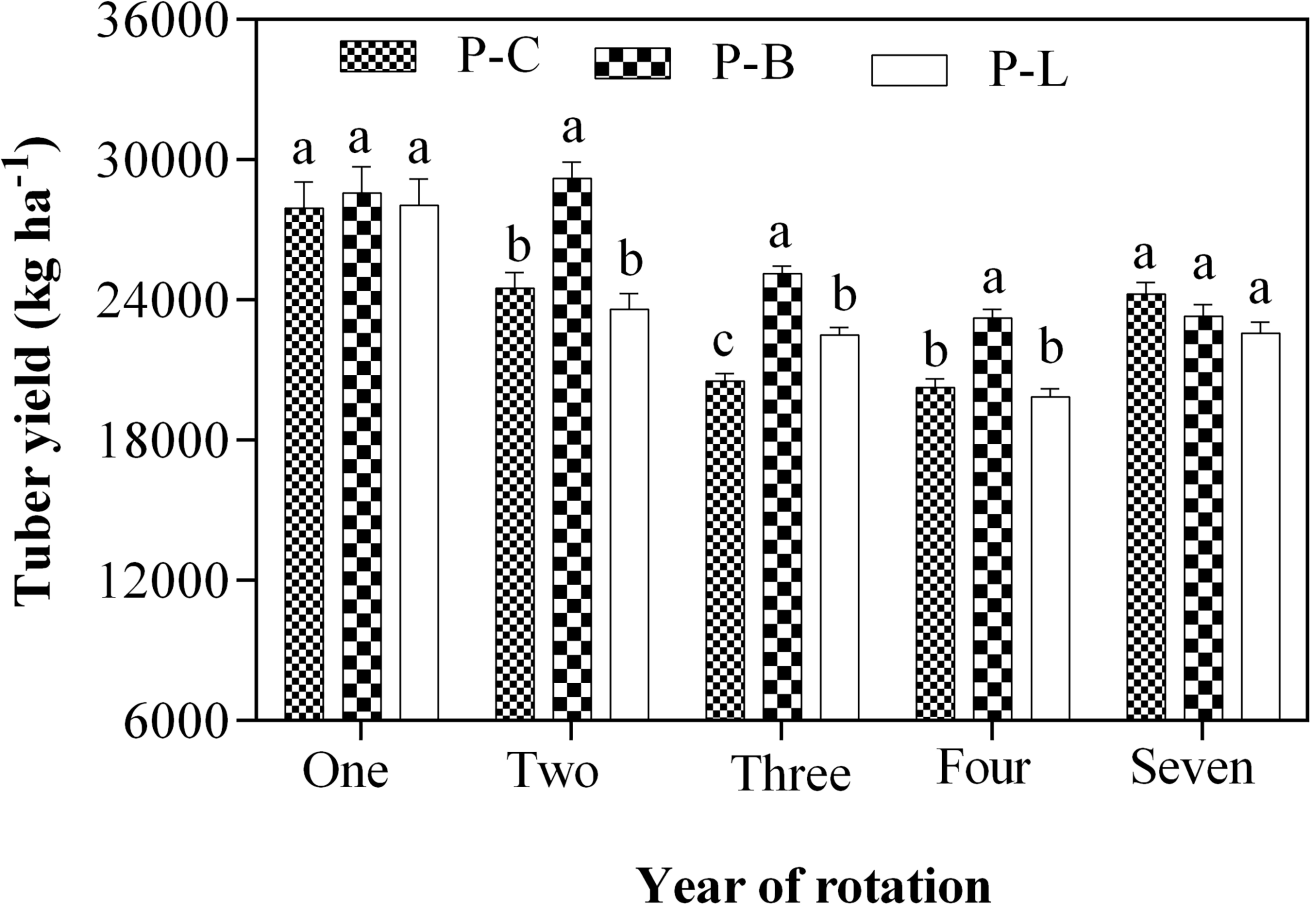
Effect of potato–legume rotation on potato tuber yield (kg ha^–1^). Different letters denote statistically different values at p<0.05. Error bars represent the SE. Mean values ± SE (n = 3), and means comparison based on Duncan’s multiple range test (p<0.05).

Overall, there were significant differences (p <0.05) in potato tuber yield depending on the treatment (Fig 4). The tuber yield under P–B rotation was significantly increased (p <0.05) in the 2–year (by 19% and 24%), 3–year (by 22% and 12%) and the 4–year (by 15% and 17%) continuous cropping soils compared to P–C and P–L rotations, respectively. In addition, P–L rotation also increased potato tuber yield by 10% compared to P–C rotation. However, no significant difference was found between treatments in the 1 and 7–year continuous cropping soils, but P–B rotation exhibited non–significantly higher tuber yield compared to the other potato–legume rotations.

### The correlation between soil microbial and enzyme activity

Results of the present study indicated significant positive correlations between BN and AA, NB (p<0.05), ANA, UR, AP (p<0.01), FN and AA, ANA, and AP (p<0.05) (Table 7). Significant positive correlations was observed between AN, NB, and UR (p<0.05). Also, significant positive correlations was found between UR and HP, and AP (p<0.01). Moreover, there were positive effects of BN, AA, NB, AP and HP (p<0.05) on potato tuber yield, while a negative correlation was observed between Y and FN (p<0.05).

**Table 7:** Correlation coefficient between soil microbial and enzyme activity.

	BN	FN	AN	AA	ANA	NB	DB	UR	AP	HP	Y
BN	1										
FN	0.28	1									
AN	0.3	0.09	1								
AA	0.48*	0.43*	0.42	1							
ANA	0.58**	0.49*	0.27	0.50*	1						
NB	0.44*	0.32	0.55*	0.53*	0.25	1					
DB	0.35	0.32	0.40	0.38	0.54*	0.07	1				
UR	0.56**	0.12	0.53*	0.53*	0.44*	0.38	0.61**	1			
AP	0.66**	0.46*	0.23	0.38	0.41	0.23	0.51*	0.71**	1		
HP	0.22	–0.07	0.40	0.39	0.51*	0.19	0.37	0.64**	0.28	1	
Y	0.50*	–0.49*	0.39	0.45*	0.41	0.46*	0.21	0.39	0.48*	0.44*	1

BN, Bacteria (10^3^ CFU /g), FN, Fungi (10^2^ CFU /g), AN, Actinomycetes (10^2^ CFU /g), AA, Aerobic azotobacter (10^2^ CFU /g), ANA, Anaerobic azotobacter (10^4^ CFU /g), NB, Nitrifying bacteria (10^3^ CFU /g), DB, Denitrifying bacteria (10^3^ CFU /g), UR, Urease (mg g^–1^), AP, Alkaline phosphatase (mg g^–1^), HP, Hydrogen peroxidase (ml g^–1^), Y, Tuber yield (Kg/ha).

* and **: statistically significant at P < 0.05 and P < 0.01, respectively.

## Discussion

Soil microorganisms are important components of the terrestrial ecosystems and their quantities can reflect not only the degree of energy metabolism, but also soil fertility status [38]. However, the change in soil microbial community is regarded one of the main factors of continuous cropping obstacle [39]. In our experiment, with the extension of potato continuous cropping years, soil fungi numbers gradually increased, whereas, soil bacterial quantity and Actinomycetes numbers decreased (above 2–3 years of continuous cropping) (Table 1). The findings reported here agrees with Ma et al. [40] and Ma et al. [4], who reported that soil microflora was significantly shifted from bacteria type to fungi type with continuous cropping.

Reasonable rotation system is an important basis for preventing continuous cropping obstacles and improving crop production [19]. As reported in previous research, subsequent cereal yields usually increases in legume–cereal rotations [41] due to the increased availability of mineral nitrogen provided by mineralization of legume residues [42], as well as the improvement of soil biological properties and availability of nutrients [43]. In this experiment, potato tuber yield significantly increased in the rotation system of potato–legumes–potato, but the effects of the rotation on potato tuber yield were different in continuous cropping field across years. The tuber yield was higher under the P–B rotation system. These results agree with previous report by Trabelsi et al. [22], who found that crop rotation with legumes can significantly increase the productivity of the non–leguminous crops, and also increase both economic and environmental benefits. In addition, another research [5, 8] reported that legume rotation induced cereal growth and yield increases seemed to depend on the ability of the legume to suppress nematodes and to enhance early N and P availability for the subsequent cereal. At the same time, rhizosphere studies showed that the effects on soil pH and acid phosphatase activity were secondary causes for the observed growth difference between rotated cereals and continuous cereals [21, 44].

Soil microbial floras were shifted from fungi type to bacteria type by rotation of Common vetch and Medicago lupulina for 1 and 2–year continuous cropping, but fungi numbers did not increase by rotation of Longdong alfalfa at the same continuous cropping years. The amelioration was significant by rotation of Medicago lupulina and Longdong alfalfa for potato soil in 3 and 7–year continuous cropping and the improvement effect of Medicago lupulina was the best. For 4–year continuous cropping field, soil microbial floras were also shifted to bacteria type by rotation of these three legumes. However, the actinomycetes mycelium decreased for up to 4–year continuous cropping field by rotation of three legumes (Table 1). Therefore, crops grown under continuous cropping soil had similar rhizosphere microbial across crop species, whereas microbial communities from the rotation soil showed greater variability, species diversity and clustered with respect to plant species [41, 45]. A similar conclusion was also drawn by Shirokikh et al. [46] in the rhizosphere of winter rye, oats, and red clover.

Microbial physiological group of nitrogen play an important role in nitrogen transformation in soil, including aerobic azotobacter, anaerobic azotobacter, nitrifying bacteria, denitrifying bacteria and ammonifying bacteria [47]. In recent years, the physiological functional group of soil microbes is a concern to many researchers. A positive effect for soil microbial community was observed under scientific and reasonable soil management practices, such as increased functional diversity of microbial communities [48–50].

After rotation with legumes, there was difference in physiological groups of microorganisms in potato continuous cropping soil. The aerobic azotobacters of 1 and 2–year potato continuous cropping soil have a very sensitive response to legumes and its number increases greatly by rotation of legumes compared with the continuous cropping soil. However, similar results were not observed in 3–year continuous potato cropping soil. Soil anaerobic nitrogen–fixing bacteria were significantly improved in all potato continuous cropping fields by rotation with Medicago lupulina and Common vetch. Ammonification can produce ammonia in the soil, which can be converted to nitrate by nitrifying bacteria. Denitrifying bacteria usually participate in the revivification of nitrate in the soil. The existence and activities of nitrifying and denitrifying bacteria in the soil play an important role in soil fertility and plant nutrition [51]. Our research results also indicated that nitrifying and denitrifying bacteria were increased under the potato–legume rotation. Therefore, all microbial in continuous cropping soil were increased by the rotation of legumes, and the variation in increment rates were high. For instance, after rotation, the increment of aerobic azotobacter was much higher than that of anaerobic azotobacter. Also, the fungi quantity were not increased as compare to bacteria, so the F:B ratio decreased in the rotation soil. This may be interpreted that both the content of nitrate nitrogen and ammonium nitrogen and their exchange rate were increased in rotation soil [52, 53].

Soil enzymes have a close relation with soil microorganisms and its biodiversity, both of which can drive soil biological metabolic processes [54]. The results indicated that soil urease and hydrogen peroxidase activities were greatly improved after rotation of legumes, and alkaline phosphatase activities also increased with the 2–year continuous cropping by the rotation of legumes. Soil enzyme activity has a close relation with soil pH [55]. Previous studies indicated that there was a higher soil pH (mean 8.17) in potato continuous cropping soil compared to non–continuous cropping soil [7]. However, a lower soil pH was observed in legumes soil due to the higher concentration of organic acids (citric acid and fumaric acid) from legumes. A higher activity of soil urease, hydrogen peroxidase and alkaline phosphatase under pH of 7.3–7.5 has been reported [55]. Enzyme activity in long–term continuous cropping soil declined gradually even though the legume crops were rotated. This may be because soil pH was increased with the years of continuous cropping, and the remediation ability of legumes was gradually decreased [7, 55].

Current study indicated a significant positive correlation between BN and AA, NB (p<0.05), ANA, UR, AP (p<0.01), FN and AA, ANA, AP (p<0.05). These findings showed that fungi and bacteria are the major soil microorganisms responsible for N cycling processes [56]. Crenshaw et al. [57] reported that fungal rather than bacterial pathways dominate the N transformation in soils. Seo and Delaune [56] also reported that fungi could also be responsible for both nitrification and denitrification processes and dominate the microbial biomass in soils. The higher numbers of AA, ANA and NB observed in the present study are consistent with greater requirements for NO_3_^–^–N [58, 59]. Therefore, soil biochemical substances were altered by the rotation of legumes because soil chemical properties (such as soil pH and salinity) were changed.

Soil microbial communities increased significantly after rotation with legumes. Number of bacteria and fungi/bacteria ratio was greatly improved by rotation of potato–Longdong alfalfa and potato–Black medic. Thus, obstacles to sustainable production of potato in the semi–arid region of Northwestern China due to continuous cropping can be reduced by adopting potato–legume rotation cropping.

## Supporting information

S1 Table Full soil microbial results. (XLSX)

S2 Table Full soil enzyme activity results. (XLSX)

S1 Fig Full potato tuber yield results. (XLSX)

## References

[1] Qin S.H., Li L.L., Wang D., Zhang J.L., Pu Y.L., 2013 Effects of limited supplemental irrigation with catchment rainfall on rain–fed potato in semi–arid areas on the Western Loess Plateau, China. Am. J. Potato Res. 90, 33–42.

[2] Zhao H., Xiong Y.C., Li F.M., Wang R.Y., Qiang S.C., Yao T.F., Mo F., 2011 Plastic film mulch for half growing–season maximized WUE and yield of potato via moisture–temperature improvement in a semi–arid agroecosystem. Agr. Water Manage. 104: 68–78.

[3] Qin S.H., Zhang J.L., Dai H.L., Wang D., Li D.M., 2014 Effect of ridge–furrow and plastic–mulching planting patterns on yield formation and water movement of potato in a semi–arid area. Agr. Water Manage. 22, 389–394.

[4] Ma K., Zhang L., Du Q., Song N.P., 2010 Effect of potato continuous cropping on soil microorganism community structure and function. J. soil water conserv. 4, 229–233.

[5] Meng P.P., Liu X., Qiu H.Z., Zhang W.R., Zhang C.H., Wang D., Zhang J.L., Shen Q.R., 2012 Fungal population structure and its biological effect in rhizosphere soil of continuously cropped potato. Chin. J. Appl. Ecol. 23(11), 3079–3086. 23431794

[6] Aparicio V., Costa J.L., 2007 Soil quality indicators under continuous cropping systems in the Argentinean Pampas. Soil Till. Res. 96, 155–165

[7] Hu Y., Guo T.W., Zhang X.C., 2009 Effect of potato continuous cropping on soil nutrients in dry land. J. Anhui Agricul. Sci. 37(12), 5436–5439, 5610.

[8] Acosta–Martínez V., Burow G., Zobeck T.M., Allen V.G. 2010 Soil Microbial Communities and Function in Alternative Systems to Continuous Cotton. Soil Sci. Soc. Am. J. 74, 1181–1192.

[9] Allison V.J., Condron L.M., Peltzer D.A., Richardson S.J., Turner B.L.., 2007 Changes in enzyme activities and soil microbial community composition along carbon and nutrient gradients at the Franz Josef chronosequence, New Zealand. Soil Biol. Biochem. 39(7), 1770–1781.

[10] Lanzén A., Jonassen I., Øvreås L., 2013 Surprising prokaryotic and eukaryotic diversity, community structure and biogeography of ethiopian soda lakes. PLOS ONE. 8(8), 65–65. 10.1371/journal.pone.0072577 PMC3758324 24023625

[11] Li Q.F., 2006 Dynamics of the microbial flora in the liriope rhizosphere and outrhizosphere during continuous cropping cears. Chin. J. Soil Sci. 37(3), 563–565.

[12] Li C.G., Li X.M., Wang J.G., 2006 Effect of soybean continuous cropping on bulk and rhizosphere soil microbial community function. Acta Ecol. Sin. 26(4), 1144–1150.

[13] Wang H.W., Wang X.X., Lv L.X., Xiao Y., Dai C.C., 2012 Effects of applying endophytic fungi on the soil biological characteristics and enzyme activities under continuously cropped peanut. Chin. J. Appl. Ecol. 23(10), 2693–2700. 23359928

[14] Qiu Y., Wang Y.J., Xie Z.K., Zhang Y.J., 2011 Temporal effects of gravel–sand mulching on soil microbial populations and soil enzyme activity in croplands with continuous cultivation. Bull. Soil Water Conserv. 31(5), 65–68.

[15] Rasool N., Reshi Z.A., Shah M.A., 2014 Effect of butachlor (G) on soil enzyme activity. Eur. J. Soil Biol. 61, 94–100.

[16] Nelson K.L., Lynch D.H., Boiteau G., 2009 Assessment of changes in soil health throughout organic potato rotation sequences. Agr. Ecosyst. Environ. 131, 220–228.

[17] Bending G.D., Turner M.K., Rayns F., Marx M., Wood M., 2004 Microbial and biochemical soil quality indicators and their potential for differentiating areas under contrasting agricultural management regimes. Soil biol. Biochem. 36, 1785–1792.

[18] Moulin A.P., Buckley K.E., Volkmar K., 2011 Soil quality as affected by amendments in bean–potato rotations. Can. J. Soil Sci. 91, 533–542.

[19] Xing F., Zhou J.Y., Jin Y.J., Sun L., Zhang J.F., Yue W., Bao Z., Ni N., Qian Y., 2011 History, theory and practice of pasture–crop rotation in China–A review. Acta Pratacul. Sin. 20(3), 245–255.

[20] Kong F.L., Chen F., Zhang H.L., Huang G.H., 2010 Effects of rotational tillage on soil physical properties and winter wheat yield. Trans. Chin. Soc. Agr. Engin. 8, 150–155.

[21] Gan Y., Hamel C., O’Donovan J.T., Cutforth H., Zentner R.P., Campbell C.A., Niu Y., Poppy L., 2015 Diversifying crop rotations with pulses enhances system productivity. Nature Scientific Reports 5, 14625. 10.1038/srep14625 PMC4589733 26424172

[22] Trabelsi D., Ammarb H.B., Mengoni A., Mhamdi R., 2012 Appraisal of the crop–rotation effect of rhizobial inoculation on potato cropping systems in relation to soil bacterial communities. Soil biol. Biochem. 54, 1–6.

[23] Gan Y., Liang C., Chai Q., Lemke R.L., Campbell C.A., Zentner R.P., 2014 Improving farming practices reduces the carbon footprint of spring wheat production. Nat. Comm. 5. 10.1038/ncomms6012 PMC4243251 25405548

[24] Gonza´lez–Cha´vez M.C.A., Aitkenhead–Peterson J.A., Gentry T.J., Zuberer D., Hons F., Loeppert R., 2010 Soil microbial community, C, N, and P responses to long–term tillage and crop rotation. Soil Till. Res. 106, 285–293.

[25] Jouquet P., Chintakunta S., Bottinelli N., Subramanian S., Caner L., 2016 The influence of fungus–growing termites on soil macro and micro–aggregates stability varies with soil type. Appl. Soil Ecol. 101, 117–123.

[26] Havlin J.L., Kissel D.E., Maddux L.D., Claassen M.M., Long J.H., 1990 Crop rotation and tillage effects on soil organic carbon and nitrogen. Soil Sci. Soc. Am. J. 54(2), 448–452

[27] Lee S.B., Lee C.H., Jung K.Y., Park K.D., Lee D., Kim P.J., 2009 Changes of soil organic carbon and its fractions in relation to soil physical properties in a long–term fertilized paddy. Soil Till. Res. 104, 227–232

[28] Sandoval Marco A., Stolpe Neal B., Zagal Erick M., Mardones María. 2007 The effect of crop–pasture rotations on the C, N and S contents of soil aggregates and structural stability in a volcanic soil of south–central Chile. Acta Agriculturae Scandinavica Section B–Soil and Plant Science, 57: 255–262.

[29] Studdert G.A., Echeverría H.E., Casanovas E.M., 1996 Crop–Pasture rotation for sustaining the quality and productivity of a typic argiudoll. Soil Sci. Soc. Am. J. 61(5), 1466–1472.

[30] Munkholm L.J., Heck R.J., Deen B., 2013 Long–term rotation and tillage effects on soil structure and crop yield. Soil Till. Res. 127, 85–91.

[31] Chinese Soil Taxonomy Cooperative Research Group, 1995 Chinese soil taxonomy (revised proposal). Institute of Soil Science, Chinese Academy of Sciences. Agr. Sci. Technol. Press, Beijing.

[32] Xu G H, Zheng H Y. 1986 Handbook of soil microbial analysis. Beijing: Agricultural press.

[33] Lin X G. 2010 Research principles and methods for soil microbial. Beijing: Higher Education Press, The first edition, 38–39

[34] Guang H., Zheng H.Y., 1986 Handbook of analysis of soil microorganism. Agriculture Press, Beijing.

[35] Panikov N.S., Sizova M.V., 1996 A kinetic method for estimating the biomass of microbial functional groups in soil. J. Microbiol. Meth. 24(3), 219–230.

[36] Popov A.I., Panikov N.S., 1990 A kinetic method for the measurement of the numbers of nitrifying bacteria in soil. Microbiology (Engl. Trans.) 59, 482–486.

[37] Zhou L.K., 1987 Soil enzymology. Beijing Science Press, Beijing.

[38] Glaring Mikkel A., Vester Jan K., Lylloff Jeanette E., Abu Al–Soud Waleed, Sørensen Søren J., Stougaard Peter. 2015 Microbial diversity in a permanently cold and alkaline environment in greenland. PLOS ONE, 10(4): e0124863 doi: 10.1371/journal.pone.0124863 25915866 10.1371/journal.pone.0124863 PMC4411134

[39] Larkin R.P., 2008 Relative effects of biological amendments and crop rotations on soil microbial communities and soilborne diseases of potato. Soil Biol. Biochem. 40(6), 1341–1351.

[40] Ma Y., Wei M., Wang X., 2004 Variation of microflora and enzyme activity in continuous cropping cucumber soil in solar greenhouse. J. Appl. Ecol. 15 (6), 1005–1008. 15362624

[41] Alvey S., Yang C.H., Buerkert A., Crowley D.E., 2003 Cereal/legume rotation effects on rhizosphere bacterial community structure in west African soils. Biol. Fert. Soils 37(2), 73–82

[42] Kouyaté Z., Franzluebbers K., Juo A.S.R., Lloyd R., 2000 Tillage, crop residue, legume rotation, and green manure effects on sorghum and millet yields in the semiarid tropics of Mali. Plant Soil 225(1), 141–151.

[43] Marschner P., Crowley D., Yang C. H., 2004 Development of specific rhizosphere bacterial communities in relation to plant species, nutrition and soil type. Plant Soil, 261(1), 199–208.

[44] Bürkert A., Bagayoko M., Alvey S., 2001 Causes of legume–rotation effects in increasing cereal yields across the Sudanian, Sahelian and Guinean zone of West Africa. Plant Nutr.–Food security and sustainability of agro–ecosystems 92, 972–973.

[45] Lupwayi N.Z., Rice W.A., Clayton G.W., 1998 Soil microbial diversity and community structure under wheat as influenced by tillage and crop roation. Soil Biol. Biochem. 30, 1733–1741.

[46] Shirokikh I.G., Zenova G.M., Merzaeva O.V., Lapygina E.V., Lysak L.V., 2006 Abundance and structure of actinomycete complexes in the rhizosphere of winter rye, oats, and red clover. Biol. Bull. 33 (4), 404–408. 17022483

[47] Yao T., Long R.J., Shi S.L., 2007 Populations of soil nitrogen bacteria groups in alpine steppe of different disturbed habitats in Tianzhu. Acta Pedologica Sin. 44(1), 122–129.

[48] Zak J.C., Willig M.R., Moorhead D.L., Wildman H.G., 1994 Functional diversity of microbial communities: a quantitative approach. Soil Biol. Biochem. 26 (9), 1101–1108.

[49] Moeskops B., Sukristiyonubowob, Buchana D., Sleutela S., Herawatyb L., Husenb E., Saraswatib R., Setyorinib D., Neve S.D., 2010 Soil microbial communities and activities under intensive organic and conventional vegetable farming in West Java, Indonesia. Appl. Soil Ecol. 45(2), 112–120.

[50] Liu X.L., Huang Y.M., Jiang J.S., Huang H., 2012 Function of microbial physiological group in carbon and nitrogen transformation during a swine manure–straw compost. Chin. J. Environ. Eng. 6(5), 1713–1720.

[51] Ding L.L., Qi B., Shang Z.H., Long R.J., Chen X.R., Xu C.L., Zhou Q.X., 2007 Dynamics of different soil microbial physiological groups and their relationship to soil conditions under sub–alpine grasslands vegetation in the eastern–Qilian mountain. Acta Pratacul. Sin. 16(2), 9–18

[52] Feigin A., Shearer G., Kohl D.H., Commoner B., 1973 The amount and nitrogen–15 content of nitrate in soil profiles from two central Illinois fields in a corn–soybean rotation. Soil Sci. Soc. Am. J. 38(3), 465–471.

[53] Maynard D.G., Kalra Y.P., Crumbaugh J.A., 2007 Nitrate and exchangeable ammonium nitrogen (chapter 6) Pages 71–80 in Carter M.R. and Gregorich E.G., editors. Soil sampling and methods of analysis (2nd edition). CRC Press, Taylor and Francis Group, Boca Raton, FL. 1264 p.

[54] Taylor J.P., Wilson B., Mills M.S., Burns R.G., 2002 Comparison of microbial numbers and enzymatic activities in surface soils and subsoils using various techniques. Soil Biol. Biochem. 34, 387–401.

[55] Maltais–Landry G., 2015 Legumes have a greater effect on rhizosphere properties (pH, organic acids and enzyme activity) but a smaller impact on soil P compared to other cover crops. Plant Soil 394, 139–154.

[56] Seo D.C., Delaune R.D., 2010 Fungal and bacterial mediated denitrification in wetlands: Influence of sediment redox condition. Water Res. 44, 2441–2450. doi: 10.1016/j.watres.2010.01.006 20122708 10.1016/j.watres.2010.01.006

[57] Crenshaw C.L., Lauber C., Sinsabaugh R.L., 2008 Fungal control of nitrous oxide production in semiarid grassland. Biogeochemi. 87, 17–27.

[58] Grimsby L.K., Fjørtoft K., Aune J.B., 2013 Nitrogen mineralization and energy from anaerobic digestion of jatropha press cake. Energy Sustain. Dev. 17, 35–39.

[59] Larkin R.P., Griffin T.S., Honeycutt C.W., 2010 Rotation and cover crop effects on soil–borne potato diseases, tuber yield, and soil microbial communities. Plant Dis. 94, 1491–1502. 10.1094/PDIS-03-10-0172 30743393

